# Correlation of phantom‐based and log file patient‐specific QA with complexity scores for VMAT

**DOI:** 10.1120/jacmp.v15i6.4994

**Published:** 2014-11-08

**Authors:** Christina E. Agnew, Denise M. Irvine, Conor K. McGarry

**Affiliations:** ^1^ Radiotherapy Physics Northern Ireland Cancer Centre, Belfast Health and Social Care Trust Belfast Northern Ireland UK; ^2^ Centre for Cancer Research, Queens University Belfast Northern Ireland UK

**Keywords:** VMAT, complexity, trajectory log files, MLC leaf speed, gantry speed

## Abstract

The motivation for this study was to reduce physics workload relating to patient‐specific quality assurance (QA). VMAT plan delivery accuracy was determined from analysis of pre‐ and on‐treatment trajectory log files and phantom‐based ionization chamber array measurements. The correlation in this combination of measurements for patient‐specific QA was investigated. The relationship between delivery errors and plan complexity was investigated as a potential method to further reduce patient‐specific QA workload. Thirty VMAT plans from three treatment sites — prostate only, prostate and pelvic node (PPN), and head and neck (H&N) — were retrospectively analyzed in this work. The 2D fluence delivery reconstructed from pretreatment and on‐treatment trajectory log files was compared with the planned fluence using gamma analysis. Pretreatment dose delivery verification was also carried out using gamma analysis of ionization chamber array measurements compared with calculated doses. Pearson correlations were used to explore any relationship between trajectory log file (pretreatment and on‐treatment) and ionization chamber array gamma results (pretreatment). Plan complexity was assessed using the MU/ arc and the modulation complexity score (MCS), with Pearson correlations used to examine any relationships between complexity metrics and plan delivery accuracy. Trajectory log files were also used to further explore the accuracy of MLC and gantry positions. Pretreatment 1%/1 mm gamma passing rates for trajectory log file analysis were 99.1% (98.7%–99.2%), 99.3% (99.1%–99.5%), and 98.4% (97.3%–98.8%) (median (IQR)) for prostate, PPN, and H&N, respectively, and were significantly correlated to on‐treatment trajectory log file gamma results (R=0.989,p<0.001). Pretreatment ionization chamber array (2%/2 mm) gamma results were also significantly correlated with on‐treatment trajectory log file gamma results (R=0.623,p<0.001). Furthermore, all gamma results displayed a significant correlation with MCS (R>0.57,p<0.001), but not with MU/arc. Average MLC position and gantry angle errors were 0.001±0.002mm and 0.025°±0.008° over all treatment sites and were not found to affect delivery accuracy. However, variability in MLC speed was found to be directly related to MLC position accuracy. The accuracy of VMAT plan delivery assessed using pretreatment trajectory log file fluence delivery and ionization chamber array measurements were strongly correlated with on‐treatment trajectory log file fluence delivery. The strong correlation between trajectory log file and phantom‐based gamma results demonstrates potential to reduce our current patient‐specific QA. Additionally, insight into MLC and gantry position accuracy through trajectory log file analysis and the strong correlation between gamma analysis results and the MCS could also provide further methodologies to both optimize the VMAT planning and QA process.

PACS number: 87.53.Bn, 87.55.Kh, 87.55.Qr

## I. INTRODUCTION

Volumetric‐modulated arc therapy (VMAT) offers equivalent or higher conformity to target volumes and faster delivery times compared to step and shoot or dynamic intensity‐modulated radiotherapy (IMRT).^1^, ^2^ VMAT is a system for intensity‐modulated radiotherapy treatment delivery that achieves high dose conformality by optimizing the dose rate, gantry speed, and the leaf positions of the dynamic multileaf collimator. Thus, the delivery of VMAT is inherently more complex than fixed gantry IMRT deliveries. This additional complexity has led to recommendations for additional machine‐specific commissioning tests.^3^, ^4^ However, 2D patient‐specific quality assurance (QA) methods used for IMRT delivery verification have been found adequate for verifying VMAT delivery accuracy.^5^, ^6^, ^7^ Despite this, 3D verification procedures have been adopted into routine use to readily assess the effect of the rotating gantry on plan delivery.^8^, ^9^, ^10^

Currently, QA of both IMRT and VMAT treatment plans is time‐consuming, putting significant strain on resources in terms of machine time, plan preparation, and analysis.^(11)^ There is a need to reduce physics workload, yet assure the quality of treatment deliveries is maintained. Although it is well documented that a single quality assurance check cannot detect all errors,^12^, ^13^ widespread debate has not led to a consensus on which combination of quality assurance checks would provide the most robust, efficient, economic resilience against treatment delivery errors.^14^, ^15^, ^16^ Furthermore, it has been suggested that errors detected pretreatment may not be representative of errors on‐treatment^13^, ^17^ or, indeed, be clinically relevant.^18^, ^19^, ^20^

The use of linear accelerator (linac) log files to verify VMAT delivery accuracy has been reported by several groups,^13^, ^21^, ^22^, ^23^, ^24^, ^25^ although confidence intervals by which to assess VMAT delivery accuracy (MLC speed and position, gantry speed and position and dose) for various treatment sites, are lacking in published data. Linac log file analysis has been proposed to improve efficiency of patient specific QA^13^, ^22^, ^23^, ^26^ and provide insight into machine parameters not possible with phantom‐based measurements.^6^, ^21^, ^23^, ^27^ Additionally, linac log file analysis could be used to assess actual/relative delivered dose reconstructed on the patient's anatomy using the patient's original CT image set^21^, ^24^, ^28^ or the on‐treatment CBCT image set.^(25)^ It has been suggested that linac log files, together with an independent check of the treatment planning system (TPS) dose calculation, could be an efficient, effective alternative to phantom‐based patient‐specific QA measurements.^13^, ^24^ Alternatively, others argue a phantom‐based dosimetric measurement should continue as the mainstay of QA checks.^(16)^

Assessment of plan complexity has been suggested as an alternative approach to reduce QA workload.^29^, ^30^ A measure of plan complexity that is related to delivery accuracy could identify less complex plans with a higher probability of achieving accurate delivery results. A number of complexity metrics have been proposed for step‐and‐shoot IMRT^29^, ^31^, ^32^ and, of these, the modulation complexity score (MCS), which assesses the variability of leaf positions and aperture areas between segments, was found to be most sensitive to delivery and plan parameters.^(33)^ The MCS has since been successfully adapted for assessment of planning parameters on VMAT plan complexity;^(30)^ however, the relationship between VMAT plan complexity and deliverability has not yet been explored in the literature.

This study investigated potential methods for reducing patient‐specific QA. Delivery accuracy of thirty VMAT plans, delivered using Varian TrueBeam linacs, was determined from analysis of pre‐ and on‐treatment trajectory log files and phantom‐based ionization chamber array measurements. Delivery accuracy was assessed in terms of gantry angle and leaf position accuracy and through gamma analysis of 2D fluence and 3D dose distributions. The correlation in this combination of measurements for patient‐specific QA was investigated. Additionally, the relationship between gamma analysis results and plan complexity was explored as a potential method to further reduce patient‐specific QA workload.

## II. MATERIALS AND METHODS

Thirty VMAT patient plans encompassing three treatment sites — prostate only, prostate and pelvic node (PPN), and head and neck (H&N) — were retrospectively assessed in this work. The plans selected for each treatment site were from 10 consecutive patients treated in our center. All plans had been accepted for clinical treatment. Plans were generated in Eclipse (v.10.0.28), optimized using progressive resolution optimization algorithm (PRO v.10.0.28) and the final dose calculated using AAA v.10.0.28, with a 0.25 cm calculation grid size (Varian Medical System, Plato Alto, CA). Plans were created using 6 MV photon beams, and partial or full, single or dual arcs to optimize target coverage, with 177 control points per full arc, as set by the Eclipse TPS. All plans were delivered using two matched Varian TrueBeam V1.5 linear accelerators and 120 leaf millennium MLCs (Varian Medical Systems). Plan statistics are summarized in Table 1. The MCS, which assesses plan complexity based on the variability of leaf positions, aperture area between segments, and segment weight as defined by McNiven et al.,^(29)^ was determined with each control point considered as a beam segment. From this definition,^(29)^ the MCS for a simple unmodulated field approaches 1, while the MCS for complex, highly modulated fields tends to 0. Following the VMAT patient‐specific QA protocol used in our center, the delivery of all VMAT plans was assessed pretreatment using the OCTAVIUS 4D phantom with the 729 ionization chamber array and assessment of trajectory log files (pretreatment QA). In addition, for this study, the trajectory log files recorded for every fraction for all plans over the entire course of treatment were analyzed (on‐treatment QA).

**Table 1:** Summary of plan statistics.

	*Prostate*	*PPN*	*H&N*
Number of Patients	10	10	10
Fractionation	74 Gy in 37#	74 Gy in 37#	70 Gy in 35#
Average Field Size (${\text{cm}}^{2}$)	125.7±27.8	274.1±19.1	293.5±93.4
Average Number of Arcs (mean ±1 SD)	1.1±0.3	2±0	1.75±0.5
MCS^(29)^	0.395±0.064	0.302±0.055	0.237±0.067
MU/arc	478±92	325±35	324±101

### A. Pretreatment verification: OCTAVIUS 4D

Pretreatment dose delivery accuracy was determined using the OCTAVIUS 4D phantom (PTW, Freiburg, Germany), comprising the OCTAVIUS 729 2D ionization chamber array, located in a motorized cylindrical phantom with an angular range of ±360°. The array rotates with the gantry, using an inclinometer attached to the gantry to determine the gantry angle, so that the detector array remains perpendicular to the incident beam, preventing errors due to the directional dependence of the detectors. The 2D array dose measurements were used at each gantry angle to create a 3D dose distribution. A single 3D dose delivery was then assessed for each plan with gamma analysis using VeriSoft v.5.1 analysis software (PTW, Freiburg, Germany), with a global 3%/3 mm and 2%/2 mm gamma criterion, normalized to a low‐gradient region with a 10% minimum dose threshold. The minimum gamma criterion of 2%/2 mm was selected due to inherent uncertainties in setting up a geometric phantom.

### B. Pretreatment verification: trajectory log file analysis

Trajectory log files recorded during pretreatment QA (minimum three deliveries per patient) were analyzed using in‐house software written in MATLAB V.7.7.0 (MathWorks, Natick, MA) as previously described, with each control point considered as a beam segment.^(26)^ Varian TrueBeam linacs record a single trajectory log file for each treatment arc. A single trajectory log file details the information for both MLC banks contained in the paired Clinac Dynalog files with the addition of couch position, dose‐rate, beam energy, and absolute monitor units delivered.^(23)^ Leaf positions (defined at the isocenter) and gantry angles (Varian IEC 601–2–1 scale) recorded at the first position of each control point within the trajectory log file were compared to the planned delivery parameters contained in the RT DICOM plan as described by Eq. (1) and Eq. (2), respectively. Root mean squared (RMS), average, and standard deviation of leaf position errors per arc were used to quantify MLC leaf position accuracy. From Eq. (2), for a clockwise (CW) arc, a positive gantry angle error means the gantry rotates faster than planned. Similarly for a counterclockwise (CCW) arc, a positive gantry angle error means the gantry rotates slower than planned. To remove the effect of arc direction on gantry errors, gantry errors in CCW arcs were multiplied by a factor of −1 so that all positive gantry errors reflect a gantry moving faster than planned. Gantry position errors are described using the average and standard deviation of gantry angle errors over each arc.

(1) MLC Position Error(mm)=MLC Planned Position-MLC Actual Position

(2) Gantry Angle Error(deg)=Actual Gantry Angle-Planned Gantry Angle

To further explore the mechanical delivery inaccuracies, actual MLC leaf velocity (cm/s) and gantry speed (deg/s) at each control point were approximated from trajectory log file data as described by Eq. (3), (4), respectively.

(3) $${\text{MLC}}_{n}\;\text{Velocity}\;(\text{cm}/s)=\frac{\text{ML}C_{n}\text{Position}(CP_{i})-\text{ML}C_{n}\text{Position}(CP_{i+1})}{0.02\times \text{LogFileSamples}(1\to i+1)}$$

(4) $$\text{Gantry Speed}\;(\text{deg}/s)=\frac{\text{Gantry Position}(CP_{i})-\text{Gantry Position}(CP_{i+1})}{0.02\times \text{LogFileSamples}(1\to i+1)}$$

where the numerator describes the distance travelled between two consecutive control points, *n* is an active MLC leaf and *i* is the CP number. The denominator describes the time between two consecutive control points, *0.02 s* is the time resolution of the trajectory log file data, and *LogFileSamples* is the number of trajectory log file samples per control point, typically 17 samples between ${\text{CP}}_{i}$ and ${\text{CP}}_{i+1}$, depending on the dose rate and gantry speed.

Two‐dimensional fluence delivery maps were reconstructed for each arc from the trajectory log file data as the relative sum of all control points, using the actual leaf positions and actual delivered MUs recorded at the first control point position within the trajectory log file. The 2D planned fluence maps were reconstructed using the equivalent control point data contained within the RT DICOM plan. Global gamma criteria 3%/3 mm and 1%/1 mm with a 10% minimum dose threshold were used to assess fluence delivery accuracy. The stricter 1%/1 mm gamma criterion was required to differentiate difficulties in plan deliveries.

### C. On‐treatment verification: trajectory log file analysis

All trajectory log files for every fraction over the course of each patient's treatment, totaling 1769 trajectory log files, were assessed retrospectively in comparison to the planned RT DICOM treatment delivery. Delivery parameters used for pretreatment QA, namely leaf position accuracy, gantry position accuracy, and fluence delivery accuracy, were further assessed for all arcs over all patient fractions.

### D. Statistical analysis

All results are presented as mean ±1SD, with the exception of gamma passing rate, which are presented as median (interquartile range (IQR)). Relationships between variables were explored using Pearson correlations (R). Results were deemed correlated for R>0.5. Due to the sample size n=30 for phantom‐based measurements and >1000 for trajectory log file measurements, all correlations with R>0.5 were significant at p<0.005 level.

## III. RESULTS

### A. 2D fluence and 3D dose delivery accuracy

**Figure 1:**
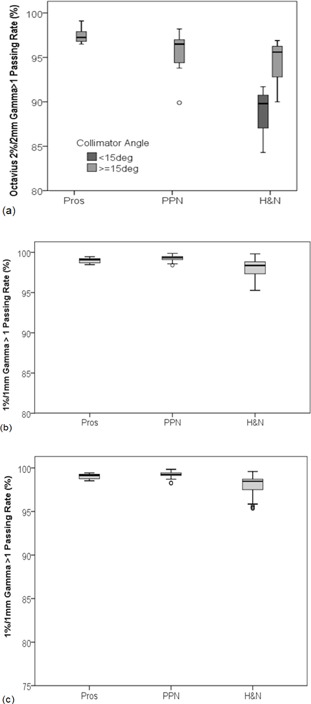
Box plots of gamma passing rates for pretreatment verification using (a) OCTAVIUS (2%/2 mm), (b) pretreatment trajectory log files (1%/1 mm), and (c) on‐treatment verification using trajectory log files (1%/1 mm). Results are stratified per treatment site ‐ prostate (Pros), prostate and pelvic node (PPN), and head and neck (H&N). Boxes represent median and interquartile range (IQR). Whiskers represent the spread of the data. Outliers are denoted by open circles. The effect of collimator angles <15° on OCTAVIUS gamma passing rates is noted for H&N cases in Fig. 1 (a).

Results for 3D OCTAVIUS dose delivery accuracy and 2D trajectory log file fluence delivery accuracy pre‐ and on‐treatment are shown in Table 2 and presented graphically in Fig. 1. Little variation was noted in 3%/3 mm gamma analysis across treatment sites or measurement devices. Stricter 2%/2 mm gamma criteria for OCTAVIUS and 1%/1 mm for trajectory log file analysis revealed variation in delivery accuracy across treatment sites. Gamma passing rates for H&N plans were reduced on all measurement devices. H&N plans were the most complex of all treatment sites investigated in this study (MCS: 0.237±0.067). Pearson correlations revealed a relationship between MCS and OCTAVIUS 2%/2 mm gamma passing rates (R=0.654,p<0.001), 1%/1 mm trajectory log file gamma passing rates pretreatment (R=0.595,p<0.001), and on‐treatment (R=0.571,p<0.001). No significant correlations were noted for any gamma passing rates and the MU/arc. Of note, a relationship between OCTAVIUS 2%/2 mm gamma passing rate and delivery collimator angle was measured as illustrated in Fig. 1(a). This is due to an increase in the number of detectors irradiated at angles close to 45° from the normal, as previously reported.^(10)^

**Table 2:** Pretreatment gamma passing rates for the OCTAVIUS and pretreatment and on‐treatment gamma passing rates for trajectory log file analysis. Results are presented as median (IQR).

		*Prostate*		*PPN*		*H&N*	
	*Gamma*	*Median*	*Range*	*Median*	*Range*	*Median*	*Range*
Pretreatment							
OCTAVIUS	3%3mm	99.90	(99.70–100)	99.75	(99.20–99.90)	98.40	(96.98–99.70)
	2%2mm	97.25	(96.80– 98. 00)	96.50	(94.25–97.28)	90.00	(87.43–92.68)
Log Files	3%3mm	99.99	(99.97–100)	100	(99.99–100)	99.99	(99.98–100)
	1%1mm	99.09	(98.70–99.23)	99.33	(99.10–99.48)	98.37	(97.34–98.82)
On‐treatment							
Log Files	3%3mm	99.99	(99.98–100)	100	(99.99–100)	99.99	(99.98–100)
	1%1mm	99.15	(98.74–99.29)	99.25	(99.13–99.44)	98.47	(97.46–98.72)

**Figure 2:**
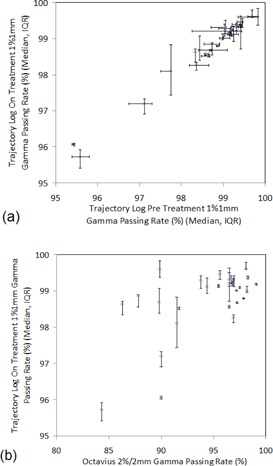
Correlation between 2D trajectory log files on‐treatment fluence delivery accuracy and (a) 2D trajectory log files pretreatment fluence delivery accuracy and (b) 3D OCTAVIUS pretreatment dose delivery accuracy. Circles represent median values and error bars represent the interquartile range (IQR).

On‐treatment trajectory log 1%/1 mm gamma results showed a strong correlation to pretreatment trajectory log 1%/1 mm gamma results (R=0.989,p<0.001), as illustrated in Fig. 2(a). The interquartile ranges of on‐treatment trajectory log gamma results were comparable to those acquired pretreatment, illustrating the consistency and reproducibility of VMAT deliveries over the course of treatment. A significant correlation was also noted between on‐treatment trajectory log fluence delivery and pretreatment OCTAVIUS dose delivery results (R=0.623,p<0.001), as presented in Fig. 2(b).

### B. MLC position accuracy

RMS MLC leaf position errors, average leaf position errors, and the standard deviation of leaf position errors were assessed per arc, per patient, and the mean and SD of these results are presented in Table 3.

**Table 3:** Summary of trajectory log file mechanical delivery errors as a function of treatment site for prostate (Pros), prostate and pelvic node (PPN), and head and neck (H&N). Data are presented as mean ±1SD.

	*Pros*	*PPN*	*H&N*
Average Leaf Errors (mm)	−0.001±0.002	0.001±0.002	0.001±0.003
Std Dev. Leaf Errors (mm)	0.134±0.009	0.162±0.009	0.138±0.012
RMS Leaf Errors (mm)	0.128±0.008	0.157±0.009	0.135±0.013
Gantry Angle Errors (deg)	0.025±0.006	0.024±0.005	0.026±0.012
Std Dev. Gantry Angle Errors (deg)	0.051±0.003	0.049±0.001	0.059±0.016

**Figure 3:**
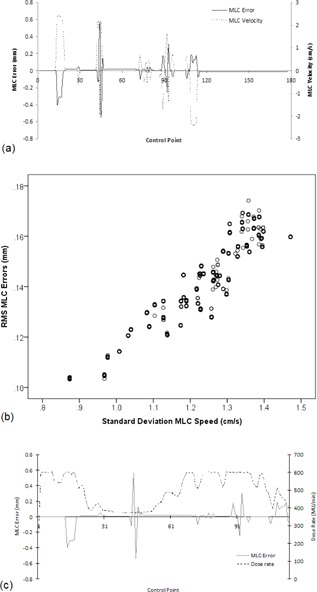
MLC leaf position errors (a) and leaf velocity for a single leaf over all control points in a sample prostate treatment arc delivery. Large transient MLC errors occur where the leaf is accelerating/decelerating. The relationship (b) between average RMS MLC error per arc, per patient, and the variability in MLC leaf speed per arc, per patient, is plotted for all 30 patients. MLC leaf position errors (c) for a single leaf and the fluctuations in dose rate over control points in the sample prostate treatment arc delivery.

Average leaf position errors were negligible, <0.005 mm over all treatment sites. However, the standard deviation and the RMS leaf position errors determined per patient illustrated that larger transient errors of up to 0.16 mm were present during the treatment delivery. Of note, the standard deviation and RMS of leaf position errors determined per patient were strongly correlated (R=0.993,p<0.001), as expected, due to the bias of both parameters to the largest errors present in the analysis. These transient leaf position errors occurred where the MLC leaves were accelerating/decelerating, as illustrated in Fig. 3(a) for a single leaf from a sample prostate plan. The relationship between MLC errors and MLC acceleration/deacceleration was also evident from the strong correlation between RMS MLC errors and the standard deviation of MLC speed (R=0.993,p<0.001) for all plans as presented in Fig. 3(b). These transient leaf position errors were not related to the fluctuations in dose rate, as illustrated in Fig. 3(c). Average, RMS, and standard deviation of leaf position errors were not related to gamma delivery results (|R|<0.3) for any measurement device. This may be due to the magnitude of these errors, of the order of 0.1 mm, while gamma analysis investigates positional errors >1 mm for trajectory log files and >2 mm in OCTAVIUS array measurements.

### C. Gantry position accuracy

**Figure 4:**
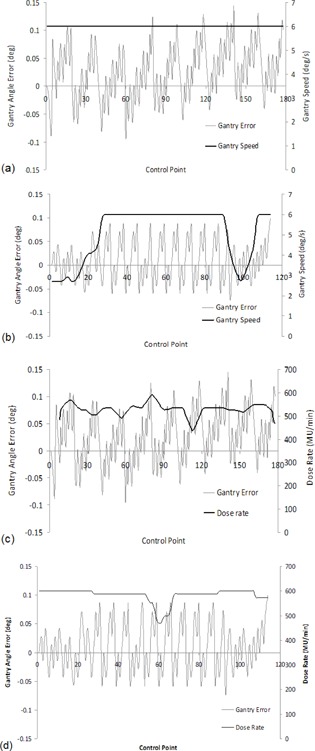
(a) and (c) illustrate gantry angle error for all control points in a sample PPN treatment arc: (a) shows with a constant gantry speed, gantry angle is corrected cyclically; (c) shows no notable relationship between gantry angle error and dose rate. (b) and (d) illustrate gantry angle error for all control points in a sample H&N treatment arc: (b) again shows where the gantry speed is constant (central region), gantry angle is corrected cyclically, while during the initial and final control points of the arc, the gantry speed varies and the gantry angle position is corrected ad‐hoc; (d) again shows no notable relationship between gantry angle error and dose rate.

Average gantry angle errors and the standard deviation of gantry angle errors were also assessed per arc, per patient, and the mean and SD of these results are presented in Table 3. Average gantry angle errors demonstrated a systematic gantry angle error of 0.025° over all treatment sites. The positive average gantry angle error indicates the gantry systematically rotated faster than planned for both CW and CCW arcs. Similar to MLC errors, the standard deviation of gantry angle errors revealed larger transient gantry angle errors were present during treatment deliveries. For deliveries with constant gantry speed, the gantry appeared to cyclically correct for position errors, as illustrated in Fig. 4(a) for a sample PPN plan and in sections of Fig. 4(b) for a sample H&N plan. With varying gantry speed, the gantry modified its position ad hoc, as illustrated in sections of Fig. 4(b). However, unlike MLC errors, this variability in gantry speed was not significantly related to variability in gantry errors (R=0.356). Additionally, the cyclic corrections of gantry position were not found to be related to variations in dose rate, as illustrated in Fig.4 (c) and (d) for either the sample PPN of H&N plans. Average and standard deviation of gantry angle errors were also not related to gamma delivery results (|R|<0.33) for all measurement devices, potentially due to the magnitude of these errors, of the order of 0.03°. The OCTAVIUS phantom has previously been found to determine the gantry angle to within 0.4° of the nominal gantry angle,^(10)^ thus detecting errors of the magnitude of 0.03° may be beyond the capabilities of the OCTAVIUS phantom. Furthermore, the accuracy of Varian C‐series DynaLog file recorded gantry angles has been found to be 0.11°, which is at the limit for data collected in this work for TrueBeam trajectory log files.^(34)^

## IV. DISCUSSION

The accuracy of VMAT deliveries on Varian TrueBeam linacs recorded by trajectory log files has been determined for three treatment sites — prostate, prostate and pelvic node, and head and neck. Although such results have previously not been published for a cohort of patients, the errors reported in this work are comparable to QA results for an example VMAT TrueBeam delivery reported elsewhere.^(23)^ Alternatively, VMAT leaf position and gantry angle errors reported with Varian C‐Series DynaLog files are an order of magnitude larger than those reported in this work with TrueBeams.^21^, ^35^, ^36^, ^37^ Furthermore, contrary to this work, previous reports with Varian C‐Series DynaLog files found the gantry rotated slower than planned.^(37)^ These differences may be a consequence of the retrospective and prospective communication utilized within the TrueBeam, rather than the retrospective communication used in the previous C‐Series linacs. This is in agreement with previously reported studies that demonstrated improvements in delivery accuracy between C‐Series linacs and TrueBeam linacs for step‐and‐shoot IMRT.^27^, ^38^

The consistency of on‐treatment trajectory log file QA results and the relationship of on‐treatment QA results with pretreatment QA results validate the use of pretreatment QA as a surrogate for on‐treatment delivery accuracy, although no pretreatment QA can mitigate against corruption of file data throughout the course of treatment. The direct correlation between trajectory log file analysis and OCTAVIUS results reveals a potential opportunity to reduce pretreatment patient specific QA. The use of linac log file pretreatment QA can ensure accurate file transfer, machine delivery capabilities and fluence delivery accuracy before treatment delivery, and precludes the need to modify any treatment parameters following pretreatment verification. Furthermore, log file QA can be performed after the first fraction to ensure no treatment parameters were modified between pretreatment QA and the first treatment delivery, which requires no physics time on the treatment machine.^13^, ^17^ However, OCTAVIUS provides a completely independent dosimetric assessment of the delivery, whereas trajectory log files recorded by the treatment machine is not completely independent and assesses the fluence rather than the dose delivery. Additionally, linac log files may not detect MLC leaf calibration errors.^(39)^ Therefore, removal of a phantom‐based measurement from pretreatment QA may be justified, but only if combined with a routine independent check of the TPS dose calculation,^(40)^ robust linac‐specific QA, and routine and independent calibration of leaf encoder positions to ensure the accuracy of linac log file data.^(39)^ In comparison to phantom‐based measurements, linac log file analysis offers significant time‐savings and greater sensitivity, and is capable of detecting delivery errors <0.1mm and <0.1°, which is not possible with phantom‐based measurements. Additionally, comparison of the linac log file with the original treatment RT DICOM maintains a direct connection with the patient treatment plan, a connection that could be broken by creation and recalculation of a phantom‐based QA plan.^(13)^

A further advantage of QA with linac log files is the potential to understand machine performance. In this study, MLC position accuracy was found to be directly related to the fluctuations in MLC speed. A relationship between gantry position errors and gantry speed was also noted, although this was not significant and gantry angle errors measured were at the known limit of Varian C‐series DynaLog file accuracy.^(34)^ Neither MLC or gantry position accuracy were related to gamma passing rates, again possibly due to the magnitude of these small errors, thus indicating these delivery errors did not combine to clinically affect the delivery accuracy. Understanding machine performance could be used to guide the planning process, with improvements in the accuracy of MLC position possible with reduction in leaf speed variability.

Previous studies have demonstrated that the MCS could be used to assess IMRT plan complexity to assist the treatment planning and optimization process,^(33)^ and improve the efficiency of the QA process by identifying less complex plans with a high probability of accurate dosimteric delivery.^(29)^ Initial work applying the MCS metric to VMAT plans found that variations in treatment planning parameters affect VMAT plan complexity.^(30)^ In this study, the MCS was also found to be related to VMAT plan deliverability measured from gamma analysis of both 2D fluence and 3D dose delivery maps. Thus the MCS, despite being developed for S&S IMRT and not considering the additional complexity in VMAT plans due to variability in MLC speed, gantry speed, and dose rate, could be used to assess the complexity of VMAT plans and, as with IMRT plans, could be a useful tool in both the planning and QA process. Furthermore, as the variability in MLC speed, but not gantry speed or dose rate, has been found to affect delivery, future work to incorporate MLC speed variability into a VMAT MCS will be undertaken to further refine this metric.

## V. CONCLUSIONS

VMAT plan delivery, recorded by trajectory log files, for three treatment sites — prostate, prostate and pelvic node, and head and neck — was highly correlated with ionization chamber array measurements, illustrating the potential to reduce the patient‐specific QA process currently utilized in our center. Trajectory log file analysis over the course of treatment was consistent, reproducible, and highly correlated with pretreatment QA measurements. Trajectory log file analysis revealed variability in MLC speed affected MLC position accuracy. Furthermore, trajectory log file and ionization chamber array gamma results were dependent on plan complexity as assessed by the MCS. Thus, a reduction in the variability of MLC speed and the use of the MCS could be utilized to further optimize the VMAT planning process and the patient‐specific QA workload.
